# Surveillance of dengue virus in individual *Aedes aegypti* mosquitoes collected concurrently with suspected human cases in Tarlac City, Philippines

**DOI:** 10.1186/s13071-020-04470-y

**Published:** 2020-11-25

**Authors:** Jean Claude Balingit, Thaddeus M. Carvajal, Mariko Saito-Obata, Maribet Gamboa, Amalea Dulcene Nicolasora, Ava Kristy Sy, Hitoshi Oshitani, Kozo Watanabe

**Affiliations:** 1 Center for Marine Environmental Studies (CMES), Ehime University, Matsuyama, Ehime Japan; 2 Graduate School of Science and Engineering, Ehime University, Matsuyama, Ehime Japan; 3 Biological Control Research Unit, Center for Natural Science and Environmental Research, De La Salle University, Taft Avenue, Manila, Philippines; 4 Department of Virology, Tohoku University Graduate School of Medicine, Sendai, Miyagi Japan; 5 Molecular Biology Laboratory, Research Institute for Tropical Medicine, Muntinlupa, Metro Manila Philippines; 6 Virology Department, Research Institute for Tropical Medicine, Muntinlupa, Metro Manila Philippines; 7 Tohoku-RITM Collaborative Research Center on Emerging and Reemerging Infectious Diseases, Muntinlupa, Metro Manila Philippines

**Keywords:** Dengue virus, Mosquito-based virus surveillance, *Aedes aegypti*, Multiplex real-time reverse transcription-polymerase chain reaction, Phylogenetic analysis, Philippines

## Abstract

**Background:**

Vector control measures are critical for the prevention and reduction of dengue virus (DENV) transmission. Effective vector control is reliant not only on knowledge of mosquito abundance, but also on the timely and accurate detection of mosquito-borne infection. Mosquito-based virus surveillance programs typically rely on pool-based mosquito testing, although whether individual-based mosquito testing is a feasible alternative to this has not been widely studied. Applying an individual-based mosquito testing approach, we conducted a 1-month surveillance study of DENV in adult *Aedes aegypti* mosquitoes in homes of suspected dengue patients during the 2015 peak dengue season in Tarlac City, Philippines to more accurately assess the mosquito infection rate and identify the DENV serotypes and genotypes concurrently co-circulating in mosquitoes and patients there.

**Methods:**

We performed a one-step multiplex real-time reverse transcription-polymerase chain reaction (RT-PCR) assay for the simultaneous detection and serotyping of DENV in patients and individual female *Ae. aegypti* mosquitoes. Additionally, we performed sequencing and phylogenetic analyses to further characterize the detected DENV serotypes in mosquitoes and patients at the genotype level.

**Results:**

We collected a total of 583 adult *Ae. aegypti* mosquitoes, of which we individually tested 359 female mosquitoes for the presence of DENV. Ten (2.8%) of the 359 female mosquitoes were positive for the presence of DENV. We detected DENV-1, DENV-2, and DENV-4 in the field-collected mosquitoes, which was consistent with the serotypes concurrently found in infected patients. Sequencing and phylogenetic analyses of the detected DENV serotypes based on the partial sequence of the evelope (*E*) gene revealed three genotypes concurrently present in the sampled mosquitoes and patients during the study period, namely DENV-1 genotype IV, DENV-2 Cosmopolitan genotype, and DENV-4 genotype II.

**Conclusions:**

We demonstrated the utility of a one-step multiplex real-time RT-PCR assay for the individual-based DENV surveillance of mosquitoes. Our findings reinforce the importance of detecting and monitoring virus activity in local mosquito populations, which are critical for dengue prevention and control.
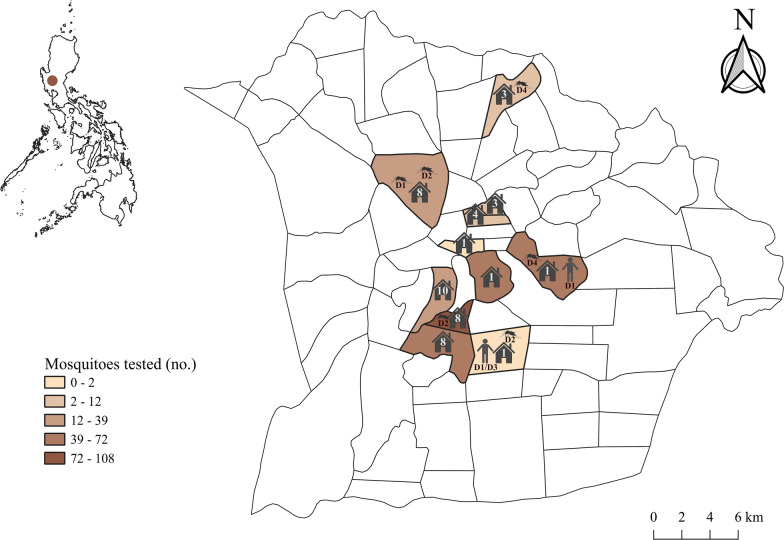

## Background

The increasing incidence and expanding geographical range of dengue virus (DENV) infections are of international concern. DENV is transmitted through a human–mosquito–human cycle throughout the tropical and subtropical regions of the world, with *Aedes aegypti* as the primary vector [1]. While isolating DENV from patients is vital for dengue disease surveillance, complementary data from mosquitoes, including viral sequences, mosquito infection rate, and serotype/genotype prevalence, can provide additional information for a better understanding of the transmission dynamics of DENV. Surveillance of the virus in field-collected mosquitoes is also useful for tracking virus activity and implementing control measures [2–5].

The detection of DENV remains a challenge owing to its low infection rate (typically approximately 0.1%) in adult female *Ae. aegypti* mosquitoes [6]. However, recent advancements in molecular virus detection techniques, particularly nucleic acid amplification tests such as reverse transcription-polymerase chain reaction (RT-PCR) and real-time RT-PCR assays, have enabled researchers to directly detect DENV RNA in field-collected mosquitoes [3–5, 7–21]. Current testing of mosquito populations for DENV serotypes has been limited to the RT-PCR of mosquito pools. Screening of mosquito pools has been widely utilized in mosquito-based virus surveillance programs owing to its cost-effectiveness, and also, in part, due to the small amount of viral RNA recovered from a single mosquito. However, one significant consideration in pooled screening is the choice of pool size, as an inappropriate pool size may lead to the inaccurate estimation of infection rates. Given that there is no consensus on the best pool size for mosquito screening, there is uncertainty concerning the accuracy of estimated minimum infection rates (MIRs) and maximal likelihood estimates (MLE) [22, 23].

An individual-based approach would be useful for monitoring the DENV infection rate with more precision. Individual-based DENV detection using RT-PCR has been reported to be technically possible when using laboratory-infected mosquitoes [7, 18]. To date, only two field studies employed an individual-based RT-PCR approach for the detection of DENV in mosquitoes [5, 8]. Utilizing an individual-based approach not only allows more accurate infection rate estimation, but also allows direct DENV RNA sequencing from a single mosquito for further genotypic characterization.

Analyzing DENV sequences from both mosquitoes and patients can potentially improve our understanding of the genetic relationships of circulating DENV serotypes. Most studies only focus on symptomatic DENV infections and do not take into account asymptomatic infections, which are increasingly contributing to the overall burden of dengue. A previous study demonstrated that asymptomatic humans infected with DENV can be infectious to mosquitoes despite their lower level of viremia [24], thereby raising the possibility of asymptomatic infections serving as a hidden reservoir for mosquito infections [25, 26], which likely leads to the dispersion of DENV. Methodologies that account for undetected infections are, therefore, warranted in dengue surveillance programs. In this context, viral data from field-collected mosquitoes have the potential to indicate asymptomatic human infections [15]. Incorporating the data of infected female *Ae. aegypti* mosquitoes into current patient-based dengue surveillance programs aids in increasing their sensitivity by enhancing the ability to predict and prevent outbreaks, as well as detect silently circulating DENV [7, 15, 19].

To this end, we conducted 1 month’s surveillance of DENV in mosquitoes collected around homes of suspected dengue patients during the 2015 peak dengue season in Tarlac City, Philippines to assess the distribution of DENV serotypes in the local mosquito population. We utilized the data on mosquito-borne DENV to serve as supporting evidence for DENV infections in patients during the same period. Our objectives were (1) to provide a more accurate estimate of DENV infection rate in mosquitoes by employing an individual-based one-step multiplex real-time RT-PCR assay, and (2) to assess the distribution of the DENV serotype and genotype circulating in mosquitoes and patients during the same period. We highlight the potential for individual-based mosquito testing for DENV surveillance, and the significance of detecting and characterizing DENV serotypes in naturally infected mosquitoes concurrent with human dengue infections for inferring local virus activity in a defined time period and area.

## Methods

### Study area

This study was conducted in Tarlac City, the provincial capital of Tarlac province located in Central Luzon, Philippines. Tarlac City is situated at the center of Tarlac province and is a densely populated urban/peri-urban area that encompasses 274.66 km^2^ of land. In 2015, the population of Tarlac City was 342,493 inhabitants [27], and the population density was 1247 inhabitants/km^2^. The city is composed of 76 *barangays* (administrative units comprising 50–100 families); of these, 19 comprise the urban area as defined by the 2000 Census of Population and Housing [28]. Maps were created using QGIS 3.6 software and edited in Inkscape (http://www.inkscape.org), with some figures created with BioRender (http://biorender.com). Data for creating the map were acquired from the Philippine geographic information system (GIS) Data website (www.philgis.org).

### Recruitment and laboratory diagnosis of patients

In 2015, there was a high prevalence of dengue fever in Tarlac City, with a total number of 1577 dengue cases (no reported deaths). Febrile inpatients in Tarlac Provincial Hospital who were suspected of being infected with DENV (onset of fever from 1 August to 31 October 2015) were recruited within 5 days of the onset of symptoms for this study. After informed consent was obtained, blood was collected and the serum was separated. The presence of the DENV NS1 antigen was initially tested using PanBio Dengue Early Rapid Kit (Alere Medical, MA) using the serum. A laboratory diagnosis of dengue fever was confirmed based on virus isolation using Vero 9013 (African green monkey) cells. Ten microliters of the serum was inoculated into the Vero 9013 cells in a Minimum Essential Medium supplemented with 10% fetal bovine serum and 100 U/ml of penicillin. Plates were incubated at 34 °C and 5% CO_2_, and infected culture fluid (ICF) was harvested after 7 and 14 days of incubation. Viral RNA was extracted from the serum and ICF using the QIAamp MinElute Virus Spin Kit (Qiagen, Hilden, Germany) based on the manufacturer’s protocol. DENV detection and serotyping were performed using a Multiplex real-time RT-PCR method [29]. The RT-PCR amplification of the DENV envelope (*E*) gene followed by sequencing was also performed to provide additional diagnostic evidence.

### Mosquito collection

The surveillance of *Ae. aegypti* mosquitoes was performed in homes of patients with suspected dengue infection from 26 August to 30 September 2015. The households of these suspected cases were categorized as follows: households of patients who tested positive for the DENV NS1 antigen using the PanBio Dengue Early Rapid kit (Alere Medical) at Tarlac Provincial Hospital during the mosquito collection period (category 1); households proximal (< 150 m) to the households of patients in category 1 (category 2); and households of suspected dengue patients reported by *barangay* health workers 15 days prior to the commencement of the mosquito collection period (category 3). For category 3, the selection of *barangays* was based on the previous epidemiological record of dengue provided by the city health office. The selected *barangays* were San Isidro, San Miguel, San Sebastian, Maliwalo, Dalayap, San Rafael, San Nicolas, Ligtasan, San Vicente, Binauganan, and Matatalaib. Based on the data of previous years, these *barangays* had a high number of reported cases. All the households gave informed consent for their voluntary participation in the mosquito surveillance. For category 1, once participants consented, mosquitoes were immediately collected within 24–48 h after positive DENV NS1 antigen result. Direct contact was made with the head of the household for house visitation and mosquito collection.

Commercially available mosquito ultraviolet (UV) light traps (Mosquito Trap: Jocanima, Metro Manila, Philippines) were used to collect mosquitoes, as previously described [30, 31]. The trap emits UV light and generates heat and CO_2_ gas via a photocatalytic reaction on the TiO_2_-coated funnel. Decoyed mosquitoes enter the trap through the capture windows and are then strongly drawn into the capture net by a strong current produced by the ventilator. The UV light traps collected mosquitoes daily from early afternoon to early morning (14:00–07:00 hours) and were installed either inside or outside the premises of the surveyed households. One mosquito trap was installed for each household. The inspection of installed mosquito traps and gathering of trapped mosquitoes were performed every morning on a daily basis (07:00–11:00 hours). Sampled mosquitoes were sorted, labeled, identified, and separated into males and females based on pictorial keys [32]. The identified *Ae. aegypti* mosquitoes were individually kept in 1.5-ml tubes containing 1.0 ml of RNAlater (Ambion, Invitrogen, CA) and stored at − 20 °C until processed.

### DENV detection in mosquitoes

Individual female mosquitoes were manually homogenized with a sterile plastic pestle in a 200 μl of 1× phosphate-buffered saline (Takara Bio, Shiga, Japan) in a 1.5-ml microcentrifuge tube. Total RNA was subsequently extracted from the homogenate using ISOGEN (Nippon Gene, Toyama, Japan), following the manufacturer’s protocol. Crude RNA was then treated with DNase using the TURBO DNA-free Kit (Ambion; Thermo Fisher Scientific, MA). DNAse-treated RNA was eluted in a 30 μl of nuclease-free molecular biology reagent water (Sigma-Aldrich, MO) and stored at − 80 °C pending analysis. The quantity and quality of the total RNA were verified for each sample with NanoDrop (Thermo Fisher Scientific).

A one-step multiplex real-time RT-PCR method [29] was adapted for DENV detection in individual *Ae. aegypti* mosquitoes. The assay was performed using the CFX96 Touch Deep Well Real-Time PCR Detection System (Bio-Rad, CA). Primer and probe sequences for DENV-2 were modified (Additional file 1: Table S1) for this protocol from the original method [29], with a few nucleotide bases either revised or deleted based on the consensus sequence of currently circulating major DENV-2 strains. Instead of Texas Red and BHQ2, the DENV-3 probe was labeled with Cy5.5 and BHQ2 (Additional file 1: Table S1). All assays were performed using the iTaq Universal Probes One-Step Kit (Bio-Rad) and conducted in 25 μl reaction mixture containing 5 μl of total RNA, 1× reaction mix, 200 nM each of DENV-1, DENV-2, DENV-3, and DENV-4 primers, and 180 nM of each probe. The one-step multiplex real-time RT-PCR assay was performed once in duplicate. The cycling conditions for all primer sets were 50 °C for 30 min, 95 °C for 2 min, followed by 45 cycles at 95 °C for 15 s and 60 °C for 1 min. Negative template controls consisted of water as a template. A sample was defined as positive if the average cycle threshold (Ct) of the sample replicates was above 15 cycles and below 37 cycles.

### DENV nucleotide sequencing

The DENV *E* gene of both mosquito (partial sequence) and patient (full-length) samples was amplified using the primers described in Additional file 2: Table S2. Briefly, reverse transcription of the total RNA using random primers was conducted using the Superscript III First-Strand Synthesis SuperMix (Invitrogen, CA), and a subsequent PCR amplification of the DENV *E* gene using the resulting cDNA as template was performed using a Phusion High-Fidelity DNA Polymerase (New England Biolabs, MA). The RT-PCR and gene-specific PCR were performed using a Bio-Rad T100 Thermal Cycler (Bio-Rad).

Amplicons were purified using the QIAquick PCR Purification Kit (Qiagen, Hilden, Germany), according to the manufacturer’s instructions. The purified PCR products of the mosquito samples were sent to Eurofins Genomics, Tokyo for Sanger sequencing. For the patient samples, cycle sequencing was performed using the BigDye Terminator v3.1 Cycle Sequencing Kit (Applied BioSystems, Foster City, CA) in a Takara PCR Thermal Cycler Dice. Sequencing reactions were purified using the BigDye XTerminator Purification Kit (Applied BioSystems) and loaded into Genetic DNA Analyzers 310, 3130, or 3730xl (Applied BioSystems). Bidirectional sequencing was performed using the primers listed in Additional file 3: Table S3 to resolve the full-length DENV *E* gene.

### DENV infection rate in mosquitoes

The number of DENV-positive mosquitoes per 1000 mosquitoes was determined from the (partial sequence) DENV *E* gene PCR and sequencing results. The infection rate was calculated as the number of DENV-positive female mosquitoes divided by the total number of female mosquitoes analyzed in the study area multiplied by 1000.

### Phylogenetic analyses

Mosquito-derived and patient-derived partial *E* gene sequences together with DENV reference sequences (Additional file 4: Table S4) were aligned using ClustalW 2.1 [33] and manually edited using Mesquite 3.3 [34]. The nucleotide sequences of the DENV isolates were submitted to GenBank database under the accession numbers MK268743–MK268752 (mosquito-derived sequences) and LC553202–LC553256 (patient-derived sequences). The phylogenetic analyses of DENV-1, DENV-2, and DENV-4 isolates were conducted using the maximum likelihood (ML) method. The best-fit substitution model was determined using the jModeltest [35] by the Bayesian information criterion. ML trees were inferred using the TN93+G parameters for DENV-1 [300 base pairs (bp)] and DENV-2 (258 bp) and the GTR+I parameter for DENV-4 (486 bp). The ML trees were constructed using PhyML 3.1 [36] and the reliability of the analyses was calculated using 1000 bootstrap replications. No outgroups were used, and DENV isolates were grouped according to genotype as previously described [37]. The trees were visualized and edited in FigTree 1.4.4 [38] and Inkscape (http://www.inkscape.org).

## Results

### Mosquito collection and DENV detection

**Fig. 1:**
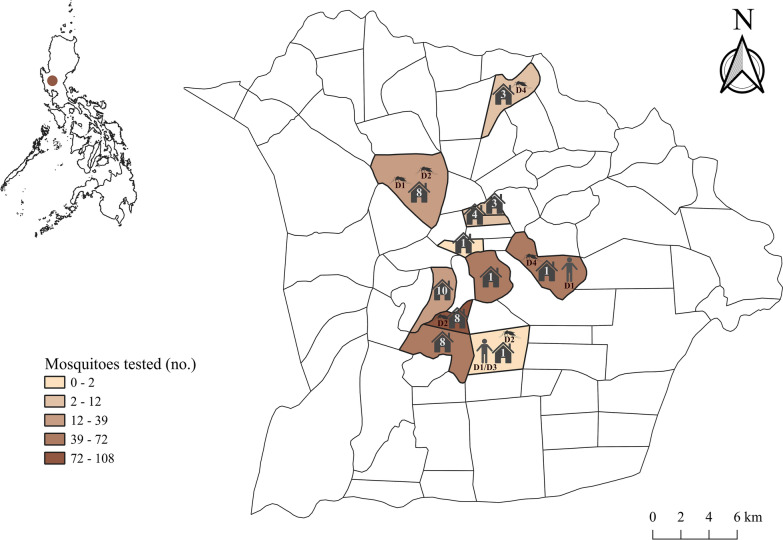
Location of Tarlac City in the Philippines (*upper left*) and choropleth map of Tarlac City. The surveyed *barangays* (*n* = 11) where female *Aedes aegypti* mosquitoes were collected are shown together with the number of surveyed homes (*n* = 48). DENV serotypes detected in mosquitoes and patients are shown on the map

In this study, 421 patients were screened for the DENV NS1 antigen at the Tarlac Provincial Hospital from August to October 2015. Of the 421 patients screened, 187 tested positive for the presence of the DENV NS1 antigen. Of these 187 patients, 32, all of whom were Tarlac City residents, tested positive in September. During the same period, mosquito surveillance was conducted around the homes of the suspected cases of dengue fever. In brief, *Ae. aegypti* mosquitoes were collected at 48 homes of households where dengue-infected mosquitoes were suspected to be present (Fig. 1). Of these 48 households, 12, 15, and 21 were grouped under categories 1, 2, and 3, respectively.

A total of 583 adult *Ae. aegypti* mosquitoes were collected, of which 383 (65.7%) were female and 200 (34.3%) were male. The average number of captured mosquitoes per trap was 1.63 ± 2.66 per day, and the highest number of captured adult *Ae. aegypti* mosquitoes in 1 day was 31. Of the 383 female mosquitoes, 359 were processed for DENV detection owing to the low quality and quantity of RNA of some of the samples. Multiplex real-time RT-PCR results were positive for 14 (3.9%) of the 359 female mosquitoes tested. RT-PCR amplification and sequencing of the DENV partial *E* gene sequence validated ten mosquito samples (2.8%) as DENV-positive (Table 1). A clear difference was noted between the Ct values of the ten validated mosquitoes and the other four mosquitoes that were not validated by RT-PCR amplification and sequencing. The Ct values for the samples that did not yield the DENV partial *E* gene sequence were ≥ 35, which was generally classified as a negative result.

**Table 1:** Dengue virus (*DENV*)-infected mosquito samples validated by reverse transcription-polymerase chain reaction (*RT-PCR*) amplification and sequencing of the partial sequence of the DENV *E* gene

Sample code	*Barangay*^a^	Household category	Total RNA concentration (ng/μl)	Mean Ct value	Detection	Validation		Mosquito DENV serotype	Patient DENV serotype
					One-step multiplex real-time RT-PCR	RT-PCR of partial *E* gene	Sequencing of partial *E* gene		
SI5-5	San Isidro	3	43.4	23.46	+	+	+	DENV-1	–
AS2-2	San Miguel	1	131.1	23.26	+	+	+	DENV-2	DENV-1/DENV-3
SI1-1	San Isidro	2	83.0	15.40	+	+	+	DENV-2	–
SI6-2	San Isidro	3	8.1	34.66	+	–	–	–	–
SI6-3	San Isidro	3	77.1	20.69	+	+	+	DENV-2	–
SI4-4	San Isidro	3	107.5	34.76	+	–	–	–	–
SB6-6	San Sebastian	3	36.6	35.97	+	–	–	–	–
SB4-22	San Sebastian	3	7.8	35.47	+	+	+	DENV-2	–
SB4-12	San Sebastian	3	36.3	34.82	+	+	+	DENV-2	–
SB4-53	San Sebastian	3	113.6	31.23	+	+	+	DENV-2	–
SB3-30	San Sebastian	3	8.5	36.11	+	–	–	–	–
AS10-49	Maliwalo	1	32.6	29.14	+	+	+	DENV-4	DENV-1
AS10-29	Maliwalo	1	106.7	26.36	+	+	+	DENV-4	DENV-1
AS12-4	Dalayap	1	122.8	36.20	+	+	+	DENV-4	–

*Ct* Cycle threshold

^a^Administrative unit comprising 50–100 families

The DENV infection rate during the 1-month mosquito surveillance period was 27.9 DENV-infected mosquitoes per 1000 female *Ae. aegypti*. Six out of the ten DENV-positive mosquitoes harbored DENV-2, three harbored DENV-4, and one harbored DENV-1 (Table 2). No DENV-3 was detected in the analyzed mosquito samples, and there were only two cases of DENV-3 detected in patients during the study period (data not shown). Notably, four DENV-infected mosquitoes were collected from the homes of patients who tested positive for the DENV NS1 antigen, but none of the DENV serotypes detected in these mosquitoes coincided with the serotypes of the patients (Table 1).

**Table 2:** DENV detection in field-collected female *Ae. aegypti* from the homes of selected households in Tarlac City (26 August–30 September 2015)

Household category	No. of households	No. of households with DENV-positive mosquitoes	No. of female mosquitoes collected		No. of female mosquitoes analyzed	No. of DENV-positive mosquitoes	Infection rate per 1000 (%)	Serotype distribution			
								DENV-1	DENV-2	DENV-3	DENV-4
1	12	3	163		146	4	27.4	0	1	0	3
2	15	1	43		43	1	23.3	0	1	0	0
3	21	3	177		170	5	29.4	1	4	0	0
Total	48	7	383		359	10	27.9	1	6	0	3

### Phylogenetic relationships among DENV serotypes isolated from mosquitoes and patients

**Fig. 2:**
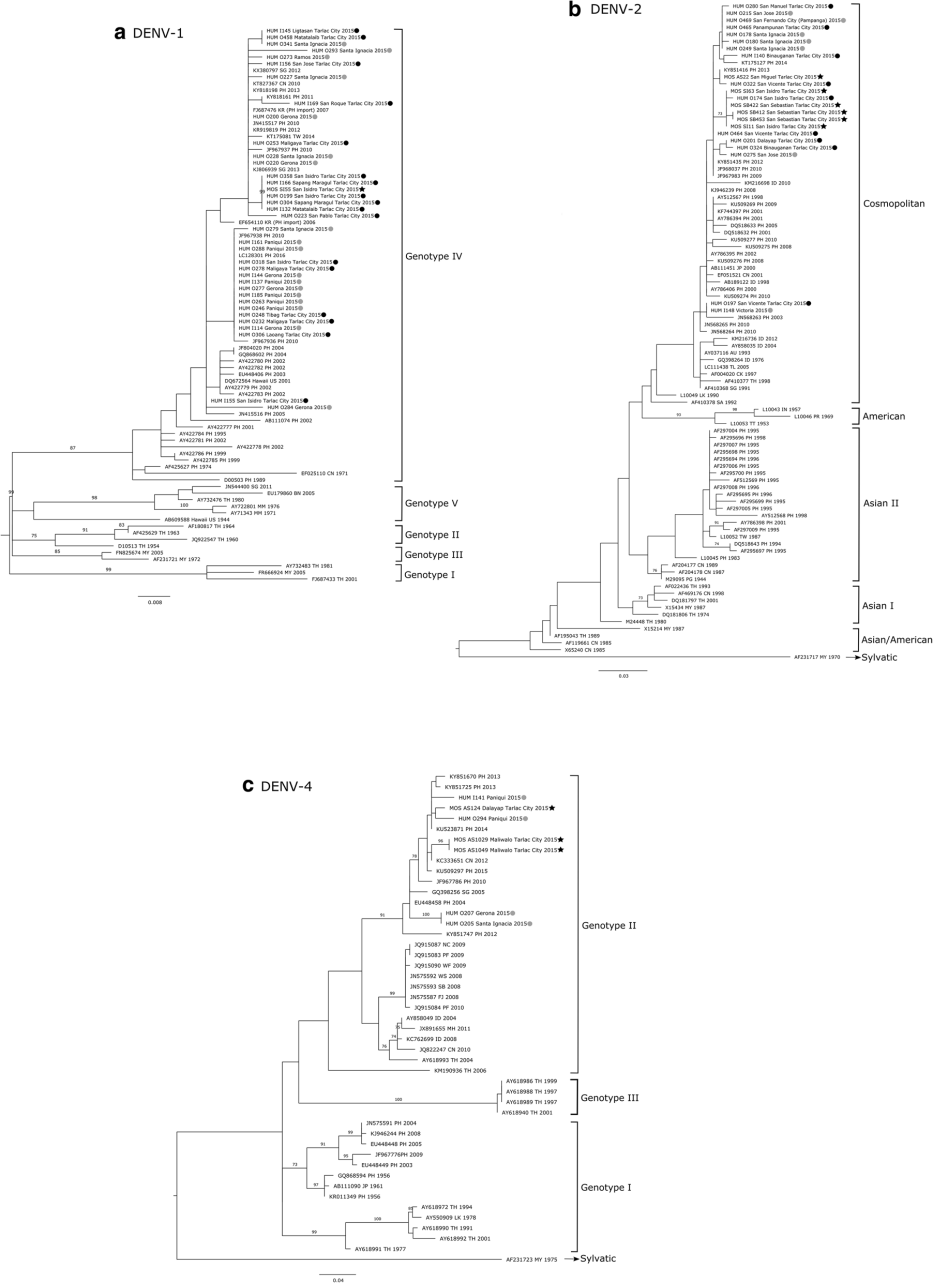
Phylogenetic tree of the partial *E* gene sequence of dengue virus (*DENV*)-1 (**a**), DENV-2 (**b**) and DENV-4 (**c**). The trees were inferred with the maximum likelihood criterion. Node support was evaluated with 1000 bootstrap replicates. Bootstrap values > 70% are shown on the* branches*. DENV sequences of mosquitoes (*black star*) and patients from Tarlac City (*black circle*) and other neighboring municipalities (*grey circle*) isolated in this study from 1 August to 31 October 2015 are included in the trees. Reference sequences are labeled by their National Center for Biotechnology Information (*NCBI*) accession numbers, two-letter International Organization for Standardization (*ISO*) country code, and corresponding year of isolation.* Scale bar* indicates nucleotide substitutions per site

Phylogenetic analysis revealed three serotypes and genotypes co-circulating in the local mosquito population during the study period, namely DENV-1 genotype IV, DENV-2 Cosmopolitan genotype, and DENV-4 genotype II (Fig. 2). Notably, the same serotypes and genotypes were present in the patient serum samples analyzed. Nucleotide identities of the DENV sequences were especially high (up to 100%) between the sampled mosquitoes and patients. High bootstrap values (70–100%) also indicated robust support for the tree topology. The DENV serotypes of the sampled mosquitoes and patients were closely related to reference strains from East Asia (China, Taiwan, Japan) and neighboring Southeast Asian countries (Indonesia and Singapore).

All DENV-1 samples from patients (*n* = 35) and mosquitoes (*n* = 1) belong to genotype IV (Fig. 2a), which is the only DENV-1 genotype reported to be circulating in the Philippines [39, 40]. DENV-2 of all mosquito (*n* = 6) and patient samples (*n* = 16) belongs to the Cosmopolitan genotype (Fig. 2b), which is currently the only circulating DENV-2 genotype in the Philippines; it was first isolated in 1998 and displaced the Asian II genotype in the early 2000s [40, 41]. Lastly, DENV-4 of all the mosquito (*n* = 3) and patient samples (*n* = 4) belongs to genotype II (Fig. 2c), which is one of the two DENV-4 genotypes currently co-circulating in the Philippines [39, 40]. The other DENV-4 genotype that has been isolated in the country is genotype I [39, 40], which was not detected in this study.

## Discussion

Mosquito-based virus surveillance is an integral component of dengue disease control as it is a significant tool for monitoring and understanding local virus activity. In this study, we show the utility of an individual-based DENV surveillance approach for the estimation of infection rate and identification of genotypes of circulating DENV serotypes in field-collected mosquitoes. We demonstrate that the DENV serotypes detected in the mosquitoes correspond with those circulating in patients, which highlights the importance of mosquito virus data for the inference of local virus activity in a defined time period and area.

The major hallmark of this study is the individual-based testing we employed for the simultaneous detection and serotyping of DENV in the RNA extracts of the field-collected mosquitoes. Previous field studies detected DENV in individual mosquitoes by using either a semi-nested RT-PCR assay [8] or a commercial duplex real-time RT-PCR dengue kit [5]. In this study, we demonstrated that a one-step multiplex real-time RT-PCR assay [29] is a potential surveillance tool for monitoring DENV in individual mosquitoes, as the method is capable of detecting all four DENV serotypes in a single mosquito in one run. This assay detects the presence of viral RNA in mosquito samples within approximately 2 h, eliminating the need to perform gel electrophoresis, as the fluorescent probes directly detect the amplified target. According to the Ct cut-off value previously reported [29], 14 mosquitoes tested positive in the one-step multiplex real-time RT-PCR assay; however, only ten of these mosquitoes were validated as positive through subsequent RT-PCR amplification and sequencing of the DENV partial *E* gene sequence. For the four mosquitoes that were not validated as DENV positive, the Ct values  were ≥ 35, which may also indicate a negative result. Alternatively, the high Ct values may have been due to low levels of DENV in the mosquitoes, which may have been below the limit of detection of the conventional RT-PCR assay used for sequencing. Thus, a real-time RT-PCR assay should be used as a screening step and not as the sole analytical method for the detection of DENV in mosquitoes. We performed serotype-specific RT-PCR amplification and sequencing of the DENV partial *E* gene sequence as confirmatory steps, thereby facilitating the direct genotypic characterization of DENV in a single mosquito.

RT-PCR amplification and sequencing of the DENV partial *E* gene sequence revealed an infection rate of 27.9 DENV-infected females per 1000 female *Ae. aegypti* mosquitoes in Tarlac City during the 1-month DENV surveillance. This infection rate is relatively high compared with the calculated (female) infection rates in previous field studies, which tested mosquito pools and performed mosquito surveillance over longer periods (Additional file 5: Table S5). The MIRs in previous studies were mainly low and varied considerably according to the pool size of mosquitoes tested (Additional file 5: Table S5). The relatively high infection rate we obtained in this study may be attributed to the targeted surveillance we implemented around residences of suspected cases of dengue. Additionally, the individual-based mosquito testing we employed might have contributed to the observed high infection rate. Individual-based mosquito testing is seldom performed in virus surveillance studies, primarily owing to logistic and financial reasons. In this study, we opted to employ an individual-based approach to more accurately estimate the infection rate in the study area. Although we were unable to assess the difference in calculated infection rates between individual-based and pool-based mosquito testing due to a limited sample volume, we argue that the sensitivity of pooled mosquito testing would be lower than that of individual mosquito testing due to a dilution effect, wherein the DENV RNA of an infected mosquito would be diluted by the RNA of uninfected mosquitoes in the pool and, as a consequence, the titer may be below the limit of detection of the test. Previous studies reported the inefficiency of pool-based infection rate indicators such as MIR and MLE for the estimation of infection rates, as these indicators are highly dependent on pool size, sample size (number of mosquitoes tested), and disease prevalence in the area examined [22, 23, 42]. For instance, MIR tends to be accurate when the level of circulating virus is low; however, during periods of high transmission, MIR generally underestimates mosquito infection [42]. Since we collected mosquitoes during the peak dengue season, using MIR would likely have led to the underestimation of the infection rate. Then again, we also need to consider the possible effect of the mosquito trapping method we used on the calculated infection rate. The abundance of mosquitoes in traps is not only affected by factors such as temperature [43], rainfall [44], and structural components of urban landscapes [45, 46], but also by the actual trapping method used. In this study, we utilized a commercial mosquito UV light trap (that generates CO_2_) because it is easy to use, can be purchased easily, is inexpensive, and can be plugged into a socket within the home where mosquitoes are being trapped. Although previous field studies have used the same trapping method [30, 31], none has definitively shown the suitability of UV light traps (baited with CO_2_) for the collection of *Ae. aegypti*. *Ae. aegypti* is a diurnal species that occupies distinct time-of-day niches and is considered to be non-specifically attracted to UV light. Thus, it is possible that the type of trapping method we used affected the number of *Ae. aegypti* that we collected. If this were true, the trapping method that we employed may have introduced a bias that affected the relationship between the estimated mosquito infection rate and the actual prevalence of infected mosquitoes [42].

Our findings showed concurrent co-circulation of similar serotypes and genotypes in mosquitoes and patients, which is similar to the results of a previous study that detected DENV in both *Aedes albopictus* and viremic patients in Catalonia, Spain [47]. The sequencing and phylogenetic analyses showed that the genotypes of the detected DENV serotypes were primarily DENV-1 genotype IV, DENV-2 Cosmopolitan genotype, and DENV-4 genotype II, suggesting hyperendemicity of dengue in Tarlac City, Philippines. Furthermore, these results are consistent with the reported multiple genotypes of DENV currently co-circulating in the Philippines [39]. No DENV-3 was detected in the mosquitoes during the study period; this may have been due to the low number of DENV-3 infected individuals in the study area in 2015 (data not shown). In the Philippines, the persistence of a single genotype of DENV-1 (genotype IV) has been shown since 1974 [40]. DENV-2, on the other hand, exhibited a genotypic shift from Asian II to the Cosmopolitan genotype in the early 2000s; the Cosmopolitan genotype has persisted ever since [40, 41], while temporal dominance of DENV-4 genotype II, with minor genotype I co-circulation, has been seen during the last 10 years [40]. Our results, therefore, suggest that there is continuous circulation of the same DENV genotypes in the Philippines, and imply that the DENV genotype distributions remain unchanged. Dengue has been classified as a notifiable disease in the Philippines since 1956 [48]. A national program directed toward community-based prevention and control was implemented nationwide in 1998 to combat dengue [49]. While a notable increase in reported cases of dengue has been observed throughout the years, the amount of published research on dengue in Philippines remains limited [50]. Moreover, to date, no report has been published on the circulating DENV serotypes and genotypes in local mosquito populations in the Philippines. To our knowledge, this is the first report of mosquito-based virus surveillance around the homes of residents with suspected infections of dengue in the Philippines. Our results underline the need for enhanced DENV surveillance to monitor DENV transmission dynamics in the Philippines.

A noteworthy finding of this study are the differences we observed between the detected DENV serotypes of mosquitoes and patients in the same home (Table 1). A study conducted in Brazil showed similar results to ours [3], and indicated that most cases of dengue were due to infection at other people’s houses or in public spaces, such as schools and workplaces [51–53]. This corroborates the notion that DENV transmission is likely driven by the movement of infected humans rather than infected mosquitoes [54, 55]. Considering the known role of asymptomatic infections in DENV transmission, these may also be of significance for these results. Members of the same household with dengue patients may be asymptomatic and may have the same serotype as mosquitoes collected in the same home. To detect asymptomatic dengue infections, members of the same household as dengue patients should also be tested for DENV [56], which is another argument for more detailed surveillance and subsequent contact tracing of dengue index cases [57].

Our study provides useful information regarding the feasibility of individual-based mosquito testing for DENV surveillance; however, some limitations should also be considered. First, the mosquito surveillance we conducted proved to be challenging owing to our limited access to the homes of patients. We only tested a small subset of mosquitoes and patients, and our findings suggest that further studies using mosquito-based virus surveillance around the homes of suspected dengue cases should employ larger sample sizes and be carried out over longer periods to fully establish the usefulness of mosquito viral data for the prevention of human cases of dengue. Moreover, our study would have provided additional information for an understanding of the dynamics of DENV transmission had the whole genome been sequenced from individual mosquitoes and patients. Owing to the limited sample volume and variations in amplification efficiency, we were only able to sequence the partial *E* gene sequence for DENV genotyping in mosquitoes. Additionally, we were not able to record the blood meal status of the mosquitoes. We indiscriminately analyzed both blood-fed and unfed mosquitoes. Detection of the virus in a blood-fed female mosquito may not indicate an actual infection but only that the mosquito has ingested viremic blood [58]. Since our results demonstrated a mismatch in the DENV serotypes of mosquitoes and patients from the same home, it is possible that the blood-fed mosquitoes had fed on the blood of asymptomatic individuals. This may represent a significant parameter for determination in future studies. Lastly, although the focus of this study was *Ae. aegypti*, it is also relevant to address the role of *Ae. albopictus* in the transmission of DENV. These mosquito species are reported to co-exist in the Philippines [59–62]. Considering the vector competence of *Ae. albopictus* for DENV [63], checking the role of this mosquito in the maintenance of DENV circulation in urban/peri-urban municipalities, like Tarlac City, is vital in future studies.

## Conclusions

In conclusion, we demonstrated that individual-based mosquito testing using a one-step multiplex real-time RT-PCR assay is a potential tool for mosquito-based DENV surveillance. Using this approach, we identified the DENV genotypes and serotypes concurrently co-circulating in mosquitoes and patients, and revealed that a high DENV infection rate was present in the local *Ae*. *aegypti* population during the 2015 peak dengue season in Tarlac City. While we have provided evidence for the continued circulation of the same DENV genotypes in the Philippines, mosquito and patient surveillance conducted in a larger population and broader setting is needed to fully understand the dynamics of circulating DENV genotypes in the Philippines. Taken together, our results reinforce the importance of DENV surveillance of field-collected mosquitoes, especially for the evaluation of local virus activity in a defined period of time and area. Phylogenetic similarity between circulating DENV serotypes in a particular geographic region may be better described by considering not only the viruses in severe cases (hospitalized patients), but also in mild cases (outpatients) and asymptomatic infections, as well as in mosquitoes.

## Supplementary information

**Additional file 1: Table S1.** Oligonucleotide primers and fluorogenic probes used in the serotype-specific dengue virus (DENV) multiplex real-time RT-PCR assay.

**Additional file 2: Table S2.** Serotype-specific primers tested for the amplification of the DENV *E* gene. Primers that were positive for partial length amplification of the *E* gene from mosquito samples are denoted by* M*, while primers positive for full-length amplification of the *E* gene from patient samples are denoted by* P*.

**Additional file 3: Table S3.** Sequencing primers used to resolve the full-length DENV *E* gene.

**Additional file 4: Table S4.** DENV-1, DENV-2 and DENV-4 global and Philippines strains used for phylogenetic analysis.

**Additional file 5: Table S5.** DENV infection rates in field-collected female *Aedes aegypti* reported in previous field studies, which utilized either reverse transcription-polymerase chain reaction (RT-PCR) or real-time RT-PCR.* MIR* Minimum infection rate,* MLE* maximum likelihood estimate of the infection rate.

## Data Availability

All data generated or analyzed during this study are included in this article and its supplementary files. All generated sequences are available from GenBank with [accession nos. MK268743–MK268752 (mosquito-derived sequences) and LC553202–LC553256 (patient-derived sequences)].

## Abbreviations

*Ae. aegypti*: *Aedes aegypti*
*Ae. albopictus*: *Aedes albopictus*
DENV: Dengue virus
cDNA: Complementary DNA
*E* gene: Envelope gene
ML: Maximum likelihood
MLE: Maximum likelihood estimation
MIR: Minimum infection rate
NS1: Nonstructural protein 1
PCR: Polymerase chain reaction
RT-PCR: Reverse transcription-polymerase chain reaction
RNA: Ribonucleic acid
UV: Ultraviolet
