# Supplementary material for: Surveillance of dengue virus in individual Aedes aegypti mosquitoes collected concurrently with suspected human cases in Tarlac City, Philippines
Source: Parasit Vectors. 2020 Nov 25;13:594. doi: 10.1186/s13071-020-04470-y (PMC7687837; doi:10.1186/s13071-020-04470-y)
Supplement: Supplementary file 1 — Additional file 1: Table S1. Oligonucleotide primers and fluorogenic probes used in the serotype-specific dengue virus (DENV) multiplex real-time RT-PCR assay. [file 13071_2020_4470_MOESM1_ESM.docx]

**Table S1.** Oligonucleotide primers and fluorogenic probes used in the serotype-specific DENV multiplex real-time RT-PCR assay.

| **DENV serotype detected** | **Primer and probes** | **Amplicon size** | **Nucleotide sequence**  **(5’ → 3’)** | **Genome position** | **Gene** | **5’ Fluorophore and**  **3’ Quencher** |
| --- | --- | --- | --- | --- | --- | --- |
| DENV-1 | DEN-1 F | 112 bp | CAAAAGGAAGTCGTGCAATA | 8973 | *NS5* | FAM/BHQ1 |
|  | DEN-1 C |  | CTGAGTGAATTCTCTCTACTGAACC | 9084 |  |  |
|  | DEN-1 probe |  | CATGTGGTTGGGAGCACGC | 8998 |  |  |
| DENV-2 | DEN-2 F***** | 78 bp | CAGG***C***TATGGCACTGTCACGAT | 1605 | *E* | HEX/BHQ1 |
|  | DEN-2 C***** |  | CCAT***T***TGCAGCAACACCATCTC | 1583 |  |  |
|  | DEN-2 probe***** |  | CTCTCCGAGAAC***G***GGCCTCGACTTCAA | 1008 |  |  |
| DENV-3 | DEN-3 F | 74 bp | GGACTGGACACACGCACTCA | 740 | *M* | CY5.5/BHQ2 |
|  | DEN-3 C |  | CATGTCTCTACCTTCTCGACTTGTCT | 813 |  |  |
|  | DEN-3 probe |  | ACCTGGATGTCGGCTGAAGGAGCTTG | 762 |  |  |
| DENV-4 | DEN-4 F | 89 bp | TTGTCCTAATGATGCTGGTCG | 904 | *M-E* | CY5/BHQ3 |
|  | DEN-4 C |  | TCCACCTGAGACTCCTTCCA | 992 |  |  |
|  | DEN-4 probe |  | TTCCTACTCCTACGCATCGCATTCCG | 960 |  |  |

*Revised primer and probe sequences. Italicized: revised nucleotide sequence; Underlined: deleted nucleotide sequence.
