# Supplementary material for: Surveillance of dengue virus in individual Aedes aegypti mosquitoes collected concurrently with suspected human cases in Tarlac City, Philippines
Source: Parasit Vectors. 2020 Nov 25;13:594. doi: 10.1186/s13071-020-04470-y (PMC7687837; doi:10.1186/s13071-020-04470-y)
Supplement: Supplementary file 2 — Additional file 2: Table S2. Serotype-specific primers tested for the amplification of the DENV E gene. Primers that were positive for partial length amplification of the E gene from mosquito samples are denoted by M, while primers positive for full-length amplification of the E gene from patient samples are denoted by P. [file 13071_2020_4470_MOESM2_ESM.docx]

**Table S2.** Serotype-specific primers tested for the amplification of the DENV *E* gene. Primers that were positive for partial length amplification of the *E* gene from mosquito samples are denoted by M, while primers positive for full-length amplification of the *E* gene from patient samples are denoted by P.

| **Serotype** | **Primers** | | **Genome Position** | **Reference** |
| --- | --- | --- | --- | --- |
| DENV-1^M^ | Fwd | D1-1229F | 1229 | Singapore NEA |
| DENV-1^M^ | Rev | D1-1710R | 1710 | Singapore NEA |
| DENV-1^P^ | Fwd | D1-820-S | 820 | Goncalvez *et al*., 2002 |
| DENV-1^P^ | Rev | D1-2600-AS | 2600 | Goncalvez *et al*., 2002 |
| DENV-1 | Fwd | D1s858 | 858 | Lanciotti *et al*., 1992 |
| DENV-1 | Rev | D1c2425 | 2425 | Lanciotti *et al*., 1992 |
| DENV-2^M,P^ | Fwd | D2F826 | 826 | Salda *et al*., 2005 |
| DENV-2^M^ | Rev | Den2-C (modified) | 1540 | Johnson *et al*., 2005 |
| DENV-2^M^ | Fwd | D2F1263 | 1263 | Salda *et al*., 2005 |
| DENV-2 | Fwd | Den-2F (modified) | 1463 | Johnson *et al*., 2005 |
| DENV-2^P^ | Rev | D2R2591 | 2591 | Salda *et al*., 2005 |
| DENV-2 | Fwd | 5’D2F798 | 798 | AFRIMS |
| DENV-2 | Fwd | 5’D2R1667 | 1667 | AFRIMS |
| DENV-2 | Fwd | Den2-1353F | 1353 | Singapore NEA |
| DENV-2 | Rev | Den2-1298R | 1298 | Singapore NEA |
| DENV-2 | Fwd | D2F1219 | 1219 | Ito *et al*., 2010 |
| DENV-2 | Fwd | Den2-835 | 835 | Wang *et al*., 2000 |
| DENV-2 | Rev | D2R1296 | 1296 | Ito *et al*., 2010 |
| DENV-3 | Fwd | 5’D3F791 | 791 | AFRIMS |
| DENV-3 | Rev | 3’D3R2492 | 2492 | AFRIMS |
| DENV-4^M^ | Fwd | 3’D4R1285 | 1285 | AFRIMS |
| DENV-4^M^ | Rev | D4CP1838 | 1838 | Lanciotti *et al*., 1992 |
| DENV-4^P^ | Fwd | D4742 | 742 | Lanciotti *et al*., 1992 |
| DENV-4 | Rev | 3’D4R1236c | 1236 | AFRIMS |
| DENV-4^P^ | Rev | D4CP2536 | 2536 | Lanciotti *et al*., 1992 |
| DENV-4 | Fwd | Den4-896F | 896 | Singapore NEA |
| DENV-4 | Rev | Den4-2434R | 2434 | Singapore NEA |
| DENV-4 | Rev | Den-4C | 992 | Johnson *et al*., 2005 |
| DENV-4 | Fwd | Den4-1760F | 1760 | Singapore NEA |
| DENV-4 | Fwd | 3’D4R1285 | 1285 | AFRIMS |
