# Supplementary material for: Surveillance of dengue virus in individual Aedes aegypti mosquitoes collected concurrently with suspected human cases in Tarlac City, Philippines
Source: Parasit Vectors. 2020 Nov 25;13:594. doi: 10.1186/s13071-020-04470-y (PMC7687837; doi:10.1186/s13071-020-04470-y)
Supplement: Supplementary file 3 — Additional file 3: Table S3. Sequencing primers used to resolve the full-length DENV E gene. [file 13071_2020_4470_MOESM3_ESM.docx]

**Table S3.** Sequencing primers used to resolve the full-length DENV *E* gene.

| **Serotype** | **Primers** | | **Genome Position** | **Reference** |
| --- | --- | --- | --- | --- |
| DENV-1 | Fwd | D1-1229F | 1229 | Singapore NEA |
| DENV-1 | Rev | 3’D1R1653 | 1653 | AFRIMS |
| DENV-1 | Fwd | D1s | 2256 | Goncalvez *et al*., 2002 |
| DENV-1 | Rev | D1-2600-AS | 2600 | Goncalvez *et al*., 2002 |
| DENV-2 | Fwd | D2F1263 | 1263 | Salda *et al*., 2005 |
| DENV-2 | Fwd | 5’D2R1667 | 1667 | AFRIMS |
| DENV-2 | Fwd | D2F1735 | 1735 | Salda *et al*., 2005 |
| DENV-2 | Rev | D2R2591 | 2591 | Salda *et al*., 2005 |
| DENV-3 | Fwd | PD31946 | 1946 | AFRIMS |
| DENV-3 | Rev | PD3R1206 | 1206 | AFRIMS |
| DENV-3 | Rev | PD3R1704 | 1704 | AFRIMS |
| DENV-3 | Rev | 3’D3R2492 | 2492 | AFRIMS |
| DENV-4 | Fwd | 3’D4R1285 | 1285 | AFRIMS |
| DENV-4 | Fwd | D4742 | 742 | Lanciotti *et al*., 1992 |
| DENV-4 | Fwd | Den4-1760F | 1760 | Singapore NEA |
| DENV-4 | Rev | 3’D4R2163 | 2163 | AFRIMS |
