# Supplementary material for: Surveillance of dengue virus in individual Aedes aegypti mosquitoes collected concurrently with suspected human cases in Tarlac City, Philippines
Source: Parasit Vectors. 2020 Nov 25;13:594. doi: 10.1186/s13071-020-04470-y (PMC7687837; doi:10.1186/s13071-020-04470-y)
Supplement: Supplementary file 4 — Additional file 4: Table S4. DENV-1, DENV-2 and DENV-4 global and Philippines strains used for phylogenetic analysis. [file 13071_2020_4470_MOESM4_ESM.docx]

**Table S4.** DENV-1, DENV-2 and DENV-4 global and Philippine strains used for phylogenetic analysis

| **Strain** | **Place of collection** | **Year of collection** | **Genotype** | **GenBank Accession no.** |
| --- | --- | --- | --- | --- |
| **DENV-1** |  |  |  |  |
| US-HI/Hawaii/1944 | Hawaii, USA | 1944 | I | AB609588 |
| ThD1_0008 81 | Thailand | 1981 | I | AY732483 |
| DENV-1/TH/BID-V2277/2001 | Thailand | 2001 | I | FJ687433 |
| MY/36000/2005 | Malaysia | 2005 | I | FR666924 |
| TH/TH-Sman/1954 | Thailand | 1954 | II | D10513 |
| DENV-1/THAI/606147 | Thailand | 1960 | II | JQ922547 |
| TH/2543-63/1963 | Thailand | 1963 | II | AF425629 |
| 16007 | Thailand | 1964 | II | AF180817 |
| MY/P72-1244/1972 | Malaysia | 1972 | III | AF231721 |
| MY/36046/2005 | Malaysia | 2005 | III | FN825674 |
| 71/02GZ | China | 1971 | IV | EF025110 |
| PRS 228682 | Philippines | 1974 | IV | AF425627 |
| Philippines 836-1 | Philippines | 1989 | IV | D00503 |
| 95SLMC28 | Philippines | 1995 | IV | AY422784 |
| 99SA236 | Philippines | 1999 | IV | AY422785 |
| 99SA660 | Philippines | 1999 | IV | AY422786 |
| US-HI/HawO3663/2001 | Hawaii, USA | 2001 | IV | DQ672564 |
| 01^St^219 | Philippines | 2001 | IV | AY422777 |
| 02SA079 | Philippines | 2002 | IV | AY422783 |
| 02SA073 | Philippines | 2002 | IV | AY422782 |
| 02SA071 | Philippines | 2002 | IV | AY422781 |
| 02SA047 | Philippines | 2002 | IV | AY422780 |
| 02SA029 | Philippines | 2002 | IV | AY422779 |
| 02RBD008 | Philippines | 2002 | IV | AY422778 |
| 02-13-1HuNIID | Philippines | 2002 | IV | AB111074 |
| D2/Philippines/0301aTw | Philippines | 2003 | IV | EU448406 |
| PH/DB007/2004 | Philippines | 2004 | IV | JF804020 |
| DENV-1/PH/BID-V2940/2004 | Philippines | 2004 | IV | GQ868602 |
| Philippines 2005 | Philippines | 2005 | IV | JN415516 |
| DenKor-07 (imported case from PH) | Philippines | 2006 | IV | EF654110 |
| DenKor-10 (imported case from PH) | Philippines | 2007 | IV | FJ687476 |
| D1/Philippines/1009bTw | Philippines | 2010 | IV | JF967937 |
| D1/Philippines/1009cTw | Philippines | 2010 | IV | JF967938 |
| D1/Philippines/1009aTw | Philippines | 2010 | IV | JF967936 |
| Philippines 2010 | Philippines | 2010 | IV | JN415517 |
| GZ/12375/D1/2010 | China | 2010 | IV | KT827367 |
| DV1/PHILIPPINES/001/2011 | Philippines | 2011 | IV | KY818161 |
| D1/SG/CT3/2012 | Singapore | 2012 | IV | KX380797 |
| Phil2012 | Philippines | 2012 | IV | KR919819 |
| DV1/PHILIPPINES/407/2013 | Philippines | 2013 | IV | KY818198 |
| SG(EHI)D1/01953Y13 | Singapore | 2013 | IV | KJ806939 |
| D1/Taiwan/820KH1409a | Taiwan | 2014 | IV | KT175081 |
| D1/Hu/Philippines/NIID13/2016 | Philippines | 2016 | IV | LC128301 |
| D1.Myanmar.40553/71 | Myanmar | 1971 | V | AY713473 |
| D1.Myanmar.40568/76 | Myanmar | 1976 | V | AY722801 |
| ThD1 0442 80 | Thailand | 1980 | V | AY732476 |
| DS06-210505 | Brunei | 2005 | V | EU179860 |
| SG(EHI)D1/22170Y11 | Singapore | 2011 | V | JN544400 |
| **DENV-2** |  |  |  |  |
| ThD2 0038 74 | Thailand | 1974 | Asian I | DQ181806 |
| M1 | Malaysia | 1987 | Asian I | X15434 |
| ThNH-52/93 | Thailand | 1993 | Asian I | AF022436 |
| GD08/98 | China | 1998 | Asian I | AF469176 |
| ThD2 0078 01 | Thailand | 2001 | Asian I | DQ181797 |
| New Guinea C | New Guinea | 1944 | Asian II | M29095 |
| D2-D80-038 | Thailand | 1980 | Asian II | M24448 |
| 2088 clone PHILIP | Philippines | 1983 | Asian II | L10045 |
| 1987 | Taiwan | 1987 | Asian II | L10052 |
| 43 | China | 1987 | Asian II | AF204178 |
| Strain 44 | China | 1989 | Asian II | AF204177 |
| Phil9406a/Tw | Philippines | 1994 | Asian II | DQ518643 |
| DOH 349 | Philippines | 1995 | Asian II | AF297006 |
| DOH 034 | Philippines | 1995 | Asian II | AF295698 |
| DOH 120 | Philippines | 1995 | Asian II | AF297004 |
| SLMC 14 | Philippines | 1995 | Asian II | AF297007 |
| SLMC 148/1995 | Philippines | 1995 | Asian II | AY512569 |
| DOH 321 | Philippines | 1995 | Asian II | AF297005 |
| DOH 078 | Philippines | 1995 | Asian II | AF295700 |
| DOH 077 | Philippines | 1995 | Asian II | AF295699 |
| SLMC 451 | Philippines | 1995 | Asian II | AF297009 |
| DOH 005 | Philippines | 1995 | Asian II | AF295697 |
| SLMC 179 | Philippines | 1996 | Asian II | AF297008 |
| BRL 020 | Philippines | 1996 | Asian II | AF295695 |
| BRL 008 | Philippines | 1996 | Asian II | AF295694 |
| PCMC 60/1998 | Philippines | 1998 | Asian II | AY512568 |
| CI-15 | Philippines | 1998 | Asian II | AF295696 |
| 01^St^193/2001 | Philippines | 2001 | Asian II | AY786398 |
| D2-04 | China | 1985 | Asian/American | X65240 |
| China isolate 04 | China | 1985 | Asian/American | AF119661 |
| M3 | Malaysia | 1987 | Asian/American | X15214 |
| D83-061 | Thailand | 1989 | Asian/American | AF195043 |
| TRIN-53 | Trinidad | 1953 | American | L10053 |
| INDIA | India | 1957 | American | L10043 |
| PR159 | Puerto Rico | 1969 | American | L10046 |
| DENV-2/ID/1046DN/1976 | Indonesia | 1976 | Cosmopolitan | GQ398264 |
| SL206 | Sri Lanka | 1990 | Cosmopolitan | L10049 |
| CAMR2 | Singapore | 1991 | Cosmopolitan | AF410368 |
| CAMR16 | Saudi Arabia | 1992 | Cosmopolitan | AF410378 |
| TSV01 | Australia | 1993 | Cosmopolitan | AY037116 |
| Cook Islands 1 | Cook Islands | 1997 | Cosmopolitan | AF004020 |
| CAMR14 | Thailand | 1998 | Cosmopolitan | AF410377 |
| 98900663 DHF DV-2 | Indonesia | 1998 | Cosmopolitan | AB189122 |
| 9D-18/1998 | Philippines | 1998 | Cosmopolitan | AY512567 |
| 00-36-1HuNIID | Japan | 2000 | Cosmopolitan | AB111451 |
| NCH57/2000 | Philippines | 2000 | Cosmopolitan | AY786406 |
| ZS01/01 | China | 2001 | Cosmopolitan | EF051521 |
| Phil0109a/Tw | Philippines | 2001 | Cosmopolitan | DQ518632 |
| 01^St^428/2001 | Philippines | 2001 | Cosmopolitan | AY786394 |
| 01-St-206 | Philippines | 2001 | Cosmopolitan | KF744397 |
| 02Sa32/2002 | Philippines | 2002 | Cosmopolitan | AY786395 |
| Philippines 2003 | Philippines | 2003 | Cosmopolitan | JN568263 |
| BA05i | Indonesia | 2004 | Cosmopolitan | AY858035 |
| Phil0509a/Tw | Philippines | 2005 | Cosmopolitan | DQ518633 |
| D2/Hu/OPD030NIID/2005 | East Timor | 2005 | Cosmopolitan | LC111438 |
| DENV2-3849 | Philippines | 2008 | Cosmopolitan | KU509275 |
| D2/Philippines/SP0261-08 | Philippines | 2008 | Cosmopolitan | KJ946239 |
| DENV2-3850 | Philippines | 2008 | Cosmopolitan | KU509276 |
| DENV2-973 | Philippines | 2009 | Cosmopolitan | KU509269 |
| D2/Philippines/0905aTw | Philippines | 2009 | Cosmopolitan | JF967983 |
| Philippines 2010b | Philippines | 2010 | Cosmopolitan | JN568265 |
| D2/Philippines/1009aTw | Philippines | 2010 | Cosmopolitan | JF968037 |
| D2/IDN/044/2010 | Indonesia | 2010 | Cosmopolitan | KM216698 |
| Philippines 2010a | Philippines | 2010 | Cosmopolitan | JN568264 |
| DENV2-14706 | Philippines | 2010 | Cosmopolitan | KU509277 |
| DENV2-3519 | Philippines | 2010 | Cosmopolitan | KU509274 |
| DV2/PHILIPPINES/296/2012 | Philippines | 2012 | Cosmopolitan | KY851435 |
| D2/IDN/Bali_108/2012 | Indonesia | 2012 | Cosmopolitan | KM216736 |
| DV2/PHILIPPINES/048/2013 | Philippines | 2013 | Cosmopolitan | KY851416 |
| D2/Philippines/1410bTw | Philippines | 2014 | Cosmopolitan | KT175127 |
| P8-1407 | Malaysia | 1970 | Sylvatic | AF231717 |
| **DENV-4** |  |  |  |  |
| DENV-4/PH/BID-V3361/1956 | Philippines | 1956 | I | GQ868594 |
| H241 | Philippines | 1956 | I | KR011349 |
| D4-61NIID | Japan | 1961 | I | AB111090 |
| ThD4 0087 77 | Thailand | 1977 | I | AY618991 |
| No.17/Sri Lanka/1978/Human | Sri Lanka | 1978 | I | AY550909 |
| ThD4 0348 91 | Thailand | 1991 | I | AY618990 |
| ThD4 0034 94 | Thailand | 1994 | I | AY618972 |
| ThD4 0485 01 | Thailand | 2001 | I | AY618992 |
| D4/Philippines/0310aTw | Philippines | 2003 | I | EU448449 |
| Philippines 2004 | Philippines | 2004 | I | JN575591 |
| D4/Philippines/0509aTw | Philippines | 2005 | I | EU448448 |
| D4/Philippines/SP0171-08 | Philippines | 2008 | I | KJ946244 |
| D4/Philippines/0909aTw | Philippines | 2009 | I | JF967776 |
| ThD4 0734 00 | Thailand | 2004 | II | AY618993 |
| D4/Philippines/0409aTw | Philippines | 2004 | II | EU448458 |
| SW36i | Indonesia | 2004 | II | AY858049 |
| DENV-4/SG/06K2270DK1/2005 | Singapore | 2005 | II | GQ398256 |
| VIROAF8 | Thailand | 2006 | II | KM190936 |
| Fiji 2008 | Fiji | 2008 | II | JN575587 |
| Samoa 2008 | Samoa | 2008 | II | JN575592 |
| Solomon Islands 2008 | Solomon Islands | 2008 | II | JN575593 |
| MKS-2139 | Indonesia | 2008 | II | KC762699 |
| WF09/010409-0001 | Wallis and Futuna | 2009 | II | JQ915090 |
| NC09/170309-6652 | New Caledonia | 2009 | II | JQ915087 |
| PF09/080409-93 | French Polynesia | 2009 | II | JQ915083 |
| PF10/150610-28 | French Polynesia | 2010 | II | JQ915084 |
| D4/Philippines/1006aTw | Philippines | 2010 | II | JF967786 |
| GZ30 | China | 2010 | II | JQ822247 |
| RMI/DB104/2011 | Marshall Islands | 2011 | II | JX891655 |
| GZ/9809/2012 | China | 2012 | II | KC333651 |
| DV4/PHILIPPINES/470/2012 | Philippines | 2012 | II | KY851747 |
| DV4/PHILIPPINES/113/2013 | Philippines | 2013 | II | KY851670 |
| DV4/PHILIPPINES/089/2013 | Philippines | 2013 | II | KY851725 |
| PH-CN08-14 | Philippines | 2014 | II | KU523871 |
| DENV4-15983 | Philippines | 2015 | II | KU509297 |
| ThD4 0476 97 | Thailand | 1997 | III | AY618988 |
| ThD4 0017 97 | Thailand | 1997 | III | AY618989 |
| TH/ThD4 0164 99 | Thailand | 1999 | III | AY618986 |
| ThD4 0439 01 | Thailand | 2001 | III | AY618940 |
| P75-514 | Malaysia | 1975 | Sylvatic | AF231723 |
