# Supplementary material for: Surveillance of dengue virus in individual Aedes aegypti mosquitoes collected concurrently with suspected human cases in Tarlac City, Philippines
Source: Parasit Vectors. 2020 Nov 25;13:594. doi: 10.1186/s13071-020-04470-y (PMC7687837; doi:10.1186/s13071-020-04470-y)
Supplement: Supplementary file 5 — Additional file 5: Table S5. DENV infection rates in field-collected female Aedes aegypti reported in previous field studies, which utilized either reverse transcription-polymerase chain reaction (RT-PCR) or real-time RT-PCR. MIR Minimum infection rate, MLE maximum likelihood estimate of the infection rate. [file 13071_2020_4470_MOESM5_ESM.docx]

**Table S5.** DENV infection rates in field-collected female *Aedes aegypti* reported in previous field studies, which utilized either RT-PCR or real-time RT-PCR. For pooled infection rates, Minimum Infection Rate is denoted as MIR, while Maximum Likelihood Estimates of the Infection Rate is denoted as MLE.

| **Reference field study** | **Country** | **Infection rate**  **(per 1000)** | **DENV serotypes detected** | **Duration** | **Detection scheme** | **DENV detection method** | **Mosquito collection method (condition of mosquito)** |
| --- | --- | --- | --- | --- | --- | --- | --- |
| Chow *et al*., 1998 | Singapore | 57.6 (MIR) | 1, 2, 3 | 16 months | Pool-based (heads only) | Semi-nested RT-PCR | Battery-operated backpack aspirators (Live, stored at -20°C) |
| Chung and Pang 2002 | Singapore | 69.1 | 1, 2, 3, 4 | 45 months | Individual (head only) | Semi-nested RT-PCR | Battery-operated backpack aspirators (Live, stored at -85°C) |
| Lourenco-de-Oliveira *et al*., 2002 | Brazil | 8.5 (MIR) | 3 | 12 months | Pool-based (whole mosquitoes) | Semi-nested RT-PCR | Manual and battery-operated backpack aspirators, and nets (Not specified, stored in liquid N_2_ at the same day of collection) |
| Urdaneta *et al*., 2005 | Venezuela | 15.9 (MIR) | 1, 3, 4 | 12 months | Pool-based (whole mosquitoes) | Semi-nested RT-PCR | Battery-operated backpack aspirators (Live, stored at -80°C) |
| Garcia-Rejon *et al*., 2008 | Mexico | 17.5 (MIR) | 1, 2, 3 | 12 months | Pool-based (whole mosquitoes) | Semi-nested RT-PCR | CDC-style backpack aspirators (Not specified, stored at -70°C) |
| Chen *et al*., 2010 | Taiwan | 1.0 (MLE) | 1, 2, 3, 4 | 12 months | Pool-based (whole mosquitoes) | One-step SYBR Green multiplex  RT-PCR | Sweeping nets (Not specified, stored at -20°C or -80°C) |
| Guedes *et al*., 2010 | Brazil | 42.2 (MIR) | 1, 2, 3 | 18 months | Pool-based (whole mosquitoes) | Semi-nested RT-PCR | Modified CDC backpack aspirators (Live, stored at -80°C) |
| Garcia-Rejon *et al*., 2011 | Mexico | 46.0 (MIR)  48.0 (MLE) | 1, 2, 3 | 4 months | Pool-based (whole mosquitoes) | Semi-nested RT-PCR | CDC-style backpack aspirators (Not specified, stored at -70°C) |
| Sanchez-Casas *et al*., 2013 | Mexico | 14.3 | 2 | 1 month | 1. Pool-based detection of DENV (pooled RNA) 2. Individual-based serotyping on positive pools | 1. Semi-nested DENV-specific RT-PCR (pooled RNA) 2. Semi-nested serotype-specific   RT-PCR (individual RNA) | CDC-style backpack aspirators (Live, stored at -70°C) |
| Mendez-Galvan *et al*., 2014 | Mexico | 3.7 | 1, 2, 4 | 3 months | 1. Pool-based detection of DENV (pooled RNA) 2. Individual-based serotyping on positive pools | 1. Semi-nested DENV-specific RT-PCR (pooled RNA) 2. Semi-nested serotype-specific   RT-PCR (individual RNA) | CDC-style backpack aspirators (Live, stored at -80°C) |
| Lau *et al*., 2015 | Malaysia | 38.0 | 1, 2, 3 | 5 months | 1. Pool-based detection of DENV (abdomens only) 2. Individual-based serotyping on positive pools (head and thorax) | 1. NS1 antigen detection kit 2. One-step TaqMan real-time   RT-PCR | Sticky traps (Not specified, stored at -20°C) |
| Peña-Garcia *et al*., 2016 | Colombia | 38.6 (MLE) | 1, 2, 3, 4 | 19 months | Pool-based (whole mosquitoes) | One-step SYBR Green multiplex  RT-PCR | Entomological nets (Not specified, stored at -70°C in RNA later®) |
| Pérez-Castro *et al*., 2016 | Colombia | 33.3 (MIR) | 1, 2, 3, 4 | 2 months | Pool-based (wings and legs removed) | Semi-nested RT-PCR | Prokopack aspirators (Not specified, stored at -80°C) |
| Medeiros *et al.*, 2018 | Brazil | 16.2 (MIR) | 1, 2, 4 | 46 months | Pool-based (whole mosquitoes) | Nested RT-PCR | Castrotrap (Live, stored at -70°C) |
| Hoyos-Lopez *et al*., 2019 | Colombia | 5.68 (MIR) | 2 | 7 years | Pool-based (whole mosquitoes) | Nested RT-PCR | CDC’s light traps bait CO_2_, manual aspirators, and Shannon traps (Dead, Triturated in minimum essential medium supplemented with 10% fetal bovine serum and 1% penicillin) |
| **This study** | **Philippines** | **27.9** | **1, 2, 4** | **1 month** | **Individual-based (whole mosquito)** | **One-step multiplex real-time RT-PCR _[29]_** | **Mosquito Trap® UV-light traps (Dead, stored at -20°C in RNAlater®)** |
